# Hydrogel-Based Drug Delivery Systems for Poorly Water-Soluble Drugs

**DOI:** 10.3390/molecules201119705

**Published:** 2015-11-13

**Authors:** Matthew McKenzie, David Betts, Amy Suh, Kathryn Bui, London Doyoung Kim, Hyunah Cho

**Affiliations:** Pharmaceutical and Administrative Sciences, St. Louis College of Pharmacy, 4588 Parkview Place, St. Louis, MO 63110, USA; Matthew.Mckenzie@stlcop.edu (M.M.); David.Betts@stlcop.edu (D.B.); Amy.Suh@stlcop.edu (A.S.); Kathryn.Bui@stlcop.edu (K.B.); Doyoung.Kim@stlcop.edu (L.D.K.)

**Keywords:** hydrogels, thermosensitive, block copolymers, hydrophobic drugs

## Abstract

Hydrogels are three-dimensional materials that can withstand a great amount of water incorporation while maintaining integrity. This allows hydrogels to be very unique biomedical materials, especially for drug delivery. Much effort has been made to incorporate hydrophilic molecules in hydrogels in the field of drug delivery, while loading of hydrophobic drugs has not been vastly studied. However, in recent years, research has also been conducted on incorporating hydrophobic molecules within hydrogel matrices for achieving a steady release of drugs to treat various ailments. Here, we summarize the types of hydrogels used as drug delivery vehicles, various methods to incorporate hydrophobic molecules in hydrogel matrices, and the potential therapeutic applications of hydrogels in cancer.

## 1. Introduction

Hydrogels are three-dimensional networks of chemically or physically cross-linked polymers that swell in water. Since the first synthetic poly(2-hydroxyethymethacrylate) hydrogels introduced by Wichterle and Lim in the late 1950s, natural and synthetic polymers with different structural features and physicochemical properties have been widely adopted to prepare “smart” or “intelligent” hydrogels as a depot-based drug delivery system to treat various diseases [1,2]. Hydrogels can be tailored to be sensitive to different environmental conditions, such as temperature, pH, and enzymatic activities at the diseased sites [3]. pH is one of the common stimulants for a successful sol-gel transformation. Hydrogels can also be temperature-sensitive: the majority of temperature-sensitive hydrogels are in a sol-state at the room temperature and undergo transformation to become a gel at a higher temperature, commonly the body temperature [4]. Hydrogels are generally biocompatible as reflected in their applications as contact lenses and as an exogenous barrier in the peritoneum [5,6]. Due to their unique physicochemical properties, hydrogels have emerged as a safe and effective depot-based drug delivery system in cancer therapy [4,7]. However, hydrogels, as a drug delivery system, have been limited to carrying hydrophilic drugs rather than hydrophobic drugs due to the limited quantity/homogeneity of loaded hydrophobic drugs in hydrogel matrices. To improve drug loading capacity, in recent studies, hydrogels have been confined into networks composed of small micelles with the average particle size of ≤200 nm [8]. Due to their micellar structure (hydrophobic core-hydrophilic shell), in matrices, hydrogels can encapsulate both hydrophilic and hydrophobic compounds to loco-regionally deliver multi-drugs in a single dose [4]. Being able to deliver hydrophobic drugs via hydrogel-based delivery systems is important, in particular, for local chemotherapy [9], noting that a large number of chemotherapeutic drug candidates possess poor water solubility and have failed to reach sufficient concentrations in injectable solutions required to exhibit therapeutic potency in preclinical studies and clinical trials [10]. Incorporation of poorly water-soluble drugs into hydrogels can enhance aqueous solubility of drugs and achieve extended release of drugs thereby increasing chances of intratumoral uptake of drugs than free drugs. In this review, we focus on hydrogels for hydrophobic drug delivery, covering the types of hydrogels used as hydrophobic drug delivery vehicles, methods to load hydrophobic drugs in hydrogel matrices, and the potential applications of hydrogels in cancer therapy. 

## 2. Types of Hydrogels

### 2.1. Thermosensitive Hydrogels

**Figure 1:**
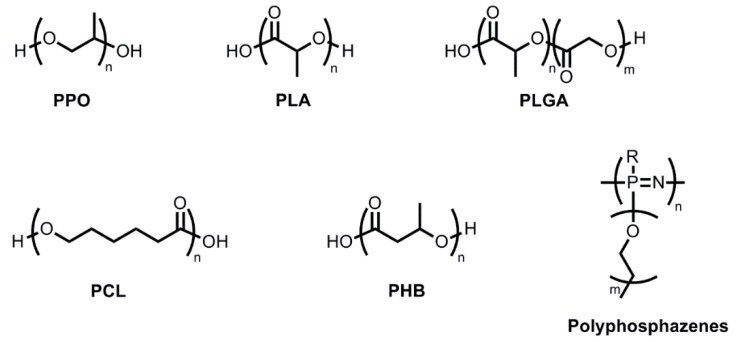
Hydrophobic polymer blocks in thermosensitive gel-forming triblock copolymers. PPO: poly(propylene oxide), PLA: (poly(d,l-lactide), PLGA: poly(d,l-lactide-co-glycolide), PCL: poly(ε-caprolactone), PHB: poly[(*R*)-3-hydroxybutyrate].

The majority of thermosensitive hydrogel-forming polymers are block copolymers containing poly(ethylene glycol) (PEG) coupled to other hydrophobic polymer blocks. These block copolymers are composed of A-blocks and B-blocks arranged as ABA or BAB. A-blocks, PEG, provide biocompatibility, low immunogenicity, and water solubility whereas B-blocks (Figure 1) including poly(propylene oxide) (PPO), polyesters such as (poly(d,l-lactide) (PLA), poly(d,l-lactide-co-glycolide) (PLGA), poly(ε-caprolactone) (PCL) and poly[(*R*)-3-hydroxybutyrate] (PHB)), polyphosphazenes, and even polypeptides, impart hydrophobicity and provide drug loading capacity for hydrophobic drugs and properties of micellization and gelation [11].

#### 2.1.1. Hydrogels with Hydrophobic PPO Blocks

Poloxamers (also known as Pluronics) are an entity widely used to create hydrogels. Poloxamers are composed of one PPO block sandwiched between two poly(ethylene oxide) (PEO) blocks. In fact, PEO-PPO-PEO triblock copolymers exist in different physical forms and vary in their properties based on molecular weights and PPO/PEO ratios of copolymers [11,12]. Poloxamers have the ability to morph into self-assembling micelles from single triblock copolymer molecules. Below the critical micelle concentration (CMC), unimeric copolymers are dispersed in water. Above the CMC, aggregation of copolymers occurs causing micellization. These triblock copolymers at a given concentration are also identifiable by their critical micelle temperature (CMT): a specific temperature at which micelles form. At the CMT, triblock copolymers form micelles. At even higher temperatures, aggregations of micelles occur due to the dehydration of both PEO and PPO blocks, leading to a phase separation [13]. Due to the unique micelle-forming structure of triblock copolymers, poloxamers have excellent thermogelling behavior; a number of reports demonstrated that dispersions of poloxamer copolymers in water are in a sol-state at lower temperatures and in a gel-state at higher temperatures [14,15,16]. The sol-gel transition has been correlated to internal changes in micellar properties, to entropic variation in the water molecules closest to the PPO segments, or to formation of a physically cross-linked, three-dimensional structure able to hold water in its network [15,17,18,19]. Overall, both micellization and gelation depend on different factors, especially temperature, polymer concentration, and PEO block length [20]. The various applications of poloxamers as a surfactant, a stabilizing agent, a suppository base, a solubilizer, a gelling agent, and an efflux pump inhibitor have been extensively studied [21]. In particular, poloxamers 407, 188, 127, and 388 have been used to deliver various compounds, such as vancomycin, clotrimazole, ciprofloxacin, 5-aminolevulinic acid, ibuprofen and even protein drugs [21]. As two hydrophilic blocks sandwiched one PPO hydrophobic block in poloxamers, the loading of hydrophobic drugs into the poloxamer-based hydrogels is limited. To improve the solubility capacity for hydrophobic compounds, alpha-cyclodextrin was used to include poloxamer-based micelles carrying payloads to form crystalline inclusion complexes called polypseudorotaxanes [21]. These inclusion complexes enable incorporation of hydrophobic compounds in polypseudorotaxanes and further modulate release properties of payloads. 

In addition to the limited loading capacity of poloxamers for hydrophobic drugs, the high CMC of poloxamers has restricted the application of poloxamers in the broad range of biomedical fields. Poloxamers have higher CMC values than many other triblock copolymers because there is a weaker hydrophobic interaction among PPO blocks requiring a greater quantity of copolymers to yield micelles and hydrogels. Hydrogels consisting of poloxamers with the high CMC values may be instable in patients. Upon the administration, a rapid physical dissociation of hydrogels (the burst effect) occurs due to the dilution of hydrogels with a substantial quantity of physiological liquids. The issues regarding the loading capacity for hydrophobic drugs, non-biodegradability, higher CMC values, and rapid dissociation of poloxamer-based hydrogels can be tackled by substituting PPO blocks with more hydrophobic and biodegradable polyesters. 

#### 2.1.2. Hydrogels with Hydrophobic PLA Blocks

Two polyesters, PLA and PLGA, as hydrophobic B-blocks of triblock copolymers have been shown to provide outstanding biodegradability, a higher gelation temperature, and extended release profiles for hydrophobic drugs [22,23,24,25,26]. Lee *et al.* synthesized PLA-PEG-PLA with acrylated groups at the ends of the PLA blocks which turned the self-assembled micelles into photo-crosslinked nanogels upon ultraviolet irradiation [22]. Photo-crosslinked PLA-PEG-PLA nanogels were 150 to 250 nm in size which can be easily manipulated by changing the concentration of crosslinkers and ultraviolet irradiation time [22]. Photo-crosslinked PLA-PEG-PLA nanogels served as a promising carrier for a hydrophobic drug, camptothecin, permitting a steady and extended release of camptothecin [22]. 

Asadi *et al.* prepared PLA-PEG-PLA hydrogels carrying a hydrophobic narcotic antagonist, naltrexone, in the size range of 128–200 nm using a different gelation technique without ultraviolet irradiation [23]. Briefly, PLA-PEG-PLA diacrylate copolymer micelles carrying naltrexone were first prepared by the nanoprecipitation method and then thermally crosslinked by heating the micelles up to 70 °C under nitrogen for 24 h. The resulting hydrogel (called nanogel in the article) suspension was further lyophilized for 48 h to remove solvents. A higher ratio of PLA/PEG blocks caused a greater naltrexone loading efficiency in PLA-PEG-PLA nanogels which theoretically provides a greater core loading capacity for hydrophobic drugs. Naltrexone incorporated in PLA-PEG-PLA nanogels using 50% of ethylene glycol dimethacrylate as a crosslinker reached <40% drug release within 35 days whereas naltrexone incorporated in PLA-PEG-PLA nanogels using 10% of ethylene glycol dimethacrylate reached 96% drug release within 15 days. This result demonstrated that PLA-PEG-PLA nanogels encapsulated a hydrophobic drug, naltrexone, in their hydrophobic core of micellar networks, and the drug release profile relied on the dissociation of crosslinked polymers. 

#### 2.1.3. Hydrogels with Hydrophobic PLGA Blocks

**Figure 2:**
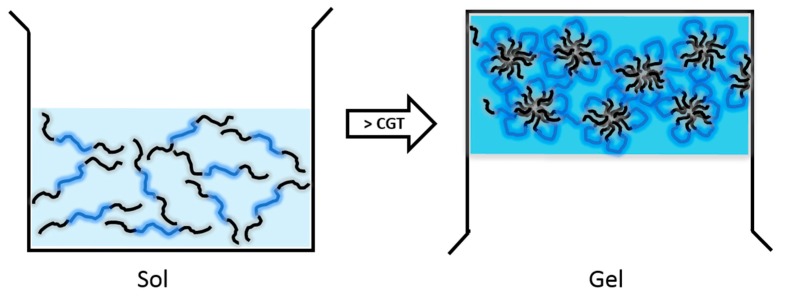
A schematic illustration of a sol-to-gel transition of PLGA-PEG-PLGA triblock copolymers. CGT: critical gelation temperature.

Given that PLA is hydrophobic, easy to crystallize, and readily forming precipitates, designing water-soluble thermogelling copolymers using PLA as hydrophobic blocks remains challenging [24]. To modify the hydrophobicity of hydrophobic blocks, lactide was copolymerized with glycolide which is more hydrophilic than lactide to yield PLGA [24]. Hydrogels with PEG and PLGA segments have been extensively investigated since 1999. One of the most popular thermosensitive products is ReGel. ReGel is a triblock copolymer comprised of PLGA and PEG arranged into PLGA-PEG-PLGA. A PLGA-PEG-PLGA triblock copolymer is a free-flowing water soluble solution (sol-state) at lower temperatures (2–15 °C) and converted from sol to gel (semi-solid gel-state) at body temperature, 37 °C [11,25,26]. At temperatures below the critical gelation temperature (CGT), as shown in Figure 2, PLGA-PEG-PLGA copolymers create loops (“flower petals”) sharing PLGA segments at the center and form “flower-like micelles” [11,25,26]. Triblock copolymers which do not contribute to the loop formation create bridges between flower-like micelles. As the temperature increases toward the CGT, hydrophobic interactions among PLGA segments increase, leading to strong micellar aggregation, loss of flowability, and inevitably gelation [11,25,26]. The gelation mechanism allows compounds with different hydrophilicity to be incorporated into the micellar matrix forming the basic structure of hydrogels. Hydrophobic drug molecules can be entrapped within the hydrophobic (PLGA-dense) regions, and hydrophilic drug molecules can be encased near the PEG regions (micellar bridging network). 

Nguyen *et al.* found that PEG-PLGA-PEG hydrogels had similar properties to ReGel [11]. Similarly, PEG-PLGA-PEG hydrogels were soluble in water at lower temperatures and gelled at higher temperatures [11]. Gelation occurred based on micellar growth/tight-packing with increasing temperature. With further increase in temperature, a sol-gel phase transition occurred due to micellar structure breakdown and dehydration of PEG and PLGA blocks. 

Jeong *et al.* proved that release profiles of drugs differ regarding the hydrophobicity of drugs incorporated in PEG-PLGA-PEG hydrogels [27]. For example, upon *in situ* injections of PEG-PLGA-PEG hydrogels carrying ketoprofen or spironolactone into a 37 °C aqueous environment, a relatively hydrophilic drug, ketoprofen (log *p* 0.97), was released in two weeks with a first-order release profile whereas spironolactone (log *p* 2.78) was released in two months with a sigmoidal release profile. This suggests that payloads incorporated in hydrogels are released based on the different drug release mechanisms: The diffusion mechanism is responsible for the release of hydrophilic drugs whereas the physical degradation/erosion of hydrogels permits release of hydrophobic drugs. 

#### 2.1.4. Hydrogels with Hydrophobic PCL, PHB, or Polyphosphazene Blocks

PCL is a semi-crystalline biodegradable polyester, which yields hydrogels with stronger integrity of gels and extended release profiles of hydrophobic drugs. Gong *et al.* synthesized PEG-PCL-PEG block copolymers and successfully prepared PEG-PCL-PEG thermosensitive hydrogels carrying a hydrophobic drug, honokiol [28]. Within 14 days, less than 50% of honokiol was gradually released from PEG-PCL-PEG hydrogels. Upon a subcutaneous injection (30% *w*/*w*, 0.5 mL) of PEG-PCL-PEG hydrogels in mice, PEG-PCL-PEG hydrogels remained at the injection site at least 14 days whereas Poloxamer 407 (Pluronic F-127) hydrogels were no longer visible at the injection site in mice on the 14th day post injection.

PHB is a polyester naturally produced by bacteria. PHB has a higher crystallinity and hydrophobicity than PLA or PCL. Due to the great hydrophobicity, copolymers carrying PHB shows a sol-gel transition with temperature change at very low concentrations (2%–5% *w*/*w*) [11]. The mechanism of gelation by micellar packing is similar to the other polyesters.

Biodegradable polyphosphazenes are a type of common thermosensitive hydrogels used today. They consist of a hydrophilic PEG block and hydrophobic amino acids, such as l-isoleucine ethyl ester (IleOEt), d,l-leucineethyl ester (LeuOEt), l-valine ethyl ester (ValOEt), or di-, tri-, and oligo-peptides in the side groups [11]. A few different studies have been performed in regards to these copolymers. One involved incorporating IleOEt with PEG which exhibited a sol-gel transition as a function of temperature change [29]. Gelation properties were modified by changing the composition of the substituents and also the molecular weight of PEG. A similar result was observed when polyphosphazenes with oligopeptides (tri- or tetra peptides) and methoxy PEG 350 as side groups exhibited a phase transition [30]. 

### 2.2. Thermo- and pH-Sensitive Hydrogels

**Figure 3:**
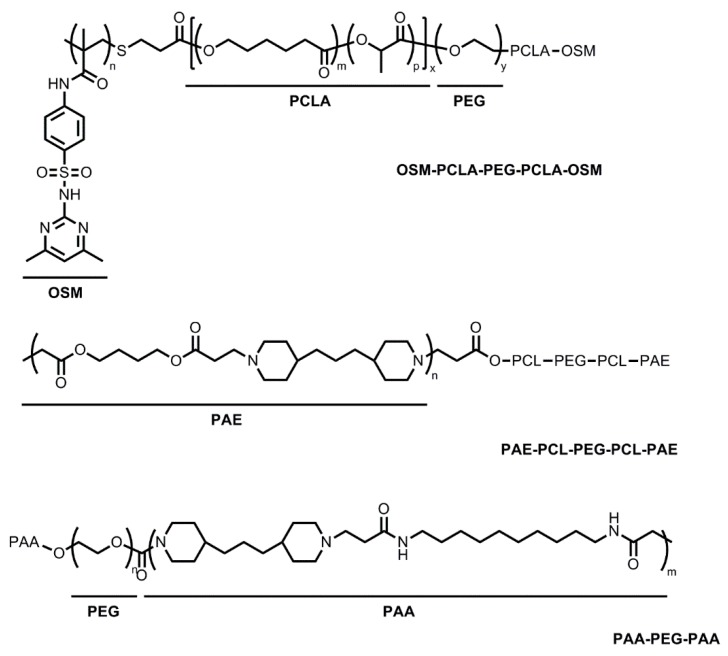
Thermo- and pH-sensitive hydrogel-forming block copolymers. OSM-PCLA-PEG-PCLA-OSM: sulfamethazine oligomers-poly(ε-caprolactone-co-lactide)-poly(ethyleneglycol)-poly(ε-caprolactone-co-lactide)-sulfamethazine oligomers, PAE-PCL-PEG-PCL-PAE: poly(*b*-aminoester)-poly(ε-caprolactone)-poly(ethylene glycol)-poly(ε-caprolactone)-poly(*b*-amino ester), PAA-PEG-PAA: poly(amidoamine)-poly(ethylene glycol)-poly(amidoamine).

Block copolymers with a pH-sensitive moiety have been added to the existing thermosensitive copolymers to overcome the following obstacles [11,31,32,33,34,35]. First, injecting thermosensitive hydrogels into the physiological environment at 37 °C tends to cause rapid gelation and blockage in the delivery syringe/needle. In addition, the lack of side chain functional groups on the thermosensitive copolymers diminishes the ability to deliver ionic species and proteins. Block copolymers containing sulfamethazine oligomers (OSMs), poly(*b*-amino ester) (PAE), and poly(amidoamine) (PAA) were able to form thermo- and pH-sensitive hydrogels, providing improved sensitivity of gelation upon the environmental changes (Figure 3).

OSMs have been used to create thermo- and pH-sensitive hydrogels. Thermosensitive poly(ε-caprolactone-co-lactide)-PEG-poly-(ε-caprolactone-co-lactide) (PCLA-PEG-PCLA) triblock copolymers were combined with OSMs to create thermo- and pH-sensitive OSM-PCLA-PEG-PCLA-OSM copolymers [31]. A thermosensitive PCLA-PEG-PCLA triblock copolymer was used as a parent molecule. PCLA-PEG-PCLA showed a sol-gel phase transition in an aqueous solution (15% *w*/*w*) as a function of temperature, however, it was not pH-sensitive [31]. Upon addition of OSMs at the endcaps, OSM-PCLA-PEG-PCLA-OSM copolymers (15% *w*/*w*) showed a sol-gel phase transition in response to both temperature and pH changes. Similarly, the gelation window grew wider with increasing pH as well as a greater PEG/PCLA ratio. It was proposed that an association of bridged micelles was responsible for the gelation of the pentablock copolymers [32]. When tested at pH 8.0 and between the temperatures of 10–70 °C, the sol-state was detected in the pentablock copolymer solution due to the ionized OSMs. However, at physiological pH of 7.4 and 15 °C, the OSMs became unionized and hence, more hydrophobic, but the pentablock copolymer still existed as in the sol-phase because of weak interactions among PCLA blocks at this lower temperature. This suggests that OSM-PCLA-PEG-PCLA-OSM hydrogels will stay in the sol-phase at pH 8.0 in the wide temperature ranges which will permit easier delivery of formulations using a syringe. In contrast, at physiologic pH of 7.4 and temperature of 37 °C, the PCLA blocks started to become hydrophobic, causing stronger hydrophobic interactions between the PCLA-OSM blocks. This led to mass micelle bridging and a gelation process ensued [31].

Basic PAE has also been used in studies to create block copolymers [33,34,35]. PAE-PCL-PEG-PCL-PAE is thermo- and pH-sensitive, forming hydrogels at pH above 6.0 with increasing temperatures [33]. Gelation was theorized to be due to micellar packing upon heating. PAE-PCL-PEG-PCL-PAE pentablock copolymers were synthesized using PCL-PEG-PCL as a parent copolymer. PCL-PEG-PCL, a parent copolymer, is sensitive to temperature change but not to pH changes. In contrast, the PAE-PCL-PEG-PCL-PAE pentablock copolymers at pH above 6.0 underwent a sol-gel phase transition in response to both temperature and pH changes [34]. The gelation window could be manipulated by changing PEG molecular weight, PAE block length, PCL/PEG ratio, and concentration of copolymers [35]. 

PAA has been shown to be both thermo- and pH-sensitive [11]. PAA-PEG-PAA triblock copolymers were in a sol-state at pH below 3.0; however, PAA-PEG-PAA triblock copolymers earned hydrophobic properties at pH above 3.0. When subjected to higher temperatures, pK_a_ of PAA-PEG-PAA copolymers decreased. PAA-PEG-PAA aqueous solutions subsequently underwent a sol-gel-to-condensed gel transition with a very high viscosity at physiological temperature and pH. The sol–gel window could be tailored by varying the polymer compositions and concentrations [11].

## 3. Preparation of Hydrogels Carrying Hydrophobic Drugs

Hydrogel systems as drug delivery vehicles have been commonly used to deliver hydrophilic compounds. More recently, studies focusing on delivery of hydrophobic drugs using hydrogel systems have been reported to improve solubility of hydrophobic drugs in water, achieve a steady release of drugs, and minimize the burst effect of drugs in patients. 

### 3.1. Simple Mixing below the CGT

One of the ways to incorporate hydrophobic drugs within a hydrogel matrix was seen in the study conducted by Almonen *et al.*, in which progesterone (P4) was incorporated into amphiphilic glycol chitin-based hydrogels [36]. Progesterone is a lipophilic female hormone, making it difficult to treat a patient without the proper vehicle. Almonen *et al.* incorporated P4 in a glycol chitin hydrogel matrix to safely and effectively administer P4 to patients via the vaginal route. Glycol chitin-based hydrogels provided an enhanced aqueous solubility of P4, was biodegradable, and made a sol-gel transition at the body temperature [36,37]. Glycol chitin, a linear polysaccharide composed of d-glucosamine and *N*-acetyl-d-glucosamine in addition to partial deacetylation of chitin, has shown to be temperature-sensitive, nontoxic, biocompatible, and biodegradable [38]. In this study, glycol chitin carried P4 in the hydrogel matrix and gradually released P4 in the vaginal environment (37 °C, pH 4.2). The pK_a_ value of the amine groups on glycol chitin backbone is *ca.* 6.84. Thus, ionization of amines takes place at the vaginal pH and induces the repulsion between polymer chains. The ionization of amines, in turn, leads to an increase in the CGT. Therefore, glycol chitin carrying P4 maintained its gel-like integrity in the vaginal environment, and is in a sol-state at the storage condition of pH 7.2 and 4 °C. Almomen *et al.* was able to successfully incorporate P4 by simply adding P4 above the level of solubility in 5% *w*/*v* glycol chitin at pH 4.2, 4 °C. Once added, the mixture was stirred for 24 h. The resulting mixture was found to contain 0.1% *w*/*v* of P4 [36]. This method, making dispersions of a payload at a greater concentration than its intrinsic solubility (*ca.* 7 μg/mL (intrinsic solubility) *vs.* 148 μg/mL), aimed to saturate extramicellar channels in hydrogels so that hydrogels release drugs in a sustained manner without altering the structure of micellar aggregates and the CGT.

In a study conducted by Xuan *et al.*, doxorubicin was loaded into a poloxamer (P407 and P188)-based hydrogel in the presence of hydrochloric acid [39]. First, P188 and P407 were completely dissolved in distilled water at 4 °C using gentle stirring and allowed to rest overnight at 4 °C. Doxorubicin and hydrochloric acid were then added the following day with gentle stirring and left to rest overnight at 4 °C to load doxorubicin in poloxamers.

### 3.2. Caging Drug-Loaded Nanoparticles or Complexes in Hydrogels

Another example of incorporation of hydrophobic drugs in a gel matrix was found in a study conducted by Kulkarni *et al.*, in which κ-carrageenan (KC) hydrogel was used to incorporate nanostructured lipid particles, known as internally self-assembled some-particles (ISAsomes) within its hydrogel matrix [40]. The purpose of this study was to find an efficient vehicle for the drug-loaded ISAsome that would allow slow drug release and maintain the intact ISAsome structure. A hydrophobic drug was first encapsulated in the ISAsome. Once encapsulated, ISAsome was incorporated into KC hydrogels followed by dehydration resulting in a thin film. This preparation decreased porosity of hydrogels and allowed the ISAsome carrying a hydrophobic drug to be released as the hydrogel liquefied. Briefly, nanostructured lipid particles were prepared and formed into emulations. Separately, a 4% KC solution was prepared by stirring KC powder in water, and heated at 60 °C for 20 min. Equal parts of lipid emulsion and KC solution were then combined and allowed to be gently stirred at 60 °C until the mixture is visually homogenized [40].

In a study conducted by Kushwaha *et al.*, camptothecin (CPT) was incorporated in a chitosan polymer matrix that had glycerol-2-phosphate (β-GP) added to the base structure. In addition, cyclodextrin was added as a cosolvent in order to increase solubility and facilitated the delivery of CPT by complexing with the drug using guest-host interactions [41]. By creating a complex, a chitosan polymer could remain a liquid at cool to room temperature and form a gel at 37 °C, remain stable at a physiological pH, and sufficiently hold the CPT-cyclodextrin payload, allowing for a slow release of the drug locally at the injection site. The preparation started with a base chitosan solution prepared by slowly adding chitosan powder to 0.1 M hydrochloric acid at room temperature, stirred for 3 h, and autoclaved to sterilize the solution. β-GP was then added (drop-wise) to the chitosan solution, and the solution was mixed for an additional 10 min at 4 °C. Once the base vehicle was made, β-cyclodextrin was slowly added at room temperature to the chitosan β-GP solution. Once that solution was homogenized, CPT was added to the solution [41].

Another use of poloxamer-based hydrogels was reported by Ju *et al.* Paclitaxel-loaded *N*-octyl-*O*-sulfate chitosan (NOSC) micelles were prepared to increase water solubility of paclitaxel up to 1000-fold. Paclitaxel-loaded NOPSC micelles were dispersed in carboxymethyl chitosan (CMCS)-conjugated poloxamer P407 gels and mixed with glutaraldehyde to create crosslinks with NH_2_ groups on CMCS [42]. The CMCS-conjugated P407 carrying paclitaxel-loaded NOSC micelles made a gel depot upon an intratumoral injection and extended retention time at tumor sites, resulting in the improved antitumor efficacy and reduction in hepatic metabolism.

### 3.3. Solvent Evaporation Method

A study was performed by Gao *et al.*, in which docetaxel (DTX) was incorporated in a hydrogel matrix comprised of PLGA-PEG-PLGA. In order to incorporate docetaxel within the PLGA-PEG-PLGA micelles, both were dissolved in acetone, mixed and dried under vacuum. Once dried, distilled water was added to form DTX-loaded micelles. Upon the intratumoral injection of DTX-loaded PLGA-PEG-PLGA micelles, PLGA-PEG-PLA formed a gel depot at the injection site at 37 °C [43].

### 3.4. Modified Nanoprecipitation Method

In a study conducted by Pillai *et al.*, folic acid-conjugated PEG acrylic polymer (FA-CLAP) hydrogel was synthesized for curcumin delivery [44]. Curcumin was loaded into the FA-CLAP hydrogels using a lyophilizaiton procedure. FA-CLAP was dissolved in water at 1 g/100 mL, and curcumin was dissolved in chloroform. The curcumin solution was added to the hydrated FA-CLAP, and the mixture was continuously vortexed and sonicated. The mixture was then lyophilized which can be rehydrated with a sterile solvent prior to the injection.

## 4. Hydrogels in Cancer Therapy

Depending on the dosage form of a medication, the results of drug release can vary by numerous degrees. Over years, researchers have been trying to create a dosage form that would provide better bioavailability and tolerability with a decrease in side effects. Hydrogels are one of the rising drug delivery systems that accommodates for these challenges today, especially with chemotherapeutic agents. First, hydrogels could improve water solubility of hydrophobic drugs enabling concurrent delivery of multiple hydrophobic drugs. OncoGel, a non-Cremophor, ReGel (PLGA-PEG-PLGA)-based formulation of paclitaxel, was designed for locoregional delivery of paclitaxel to solid tumors [45]. OncoGel as monotherapy demonstrated acceptable local tolerability and no systemic toxicity in preclinical efficacy studies. In a CRL-1666 breast cancer spinal metastases model, OncoGel increased the survival rate *vs.* the untreated control group and improved hind limb function with minimal side effect toxicity. In a MDA-MB-231 subcutaneous breast cancer-bearing mouse model, intraperitoneally administered OncoGel at 1/10 of the systemic taxol dose exhibited a similar long-term survival result as a monotherapy. OncoGel as an adjuvant to radiation therapy in an intracranial glioblastoma mouse model demonstrated improved long-term survival rate of 37.5% whereas there was no long-term survivors when treated with the radiation therapy alone. In addition, OncoGel as an adjuvant to surgery in a CRL-1666 breast cancer spinal metastases model (placement of OncoGel into the resection cavity post-surgery) delayed the onset of paresis.

Cho *et al.* prepared PLGA-PEG-PLGA thermosensitive hydrogels carrying three hydrophobic drugs, paclitaxel, 17-AAG and rapamycin (Triogel) at 6, 6, 3 mg/mL, respectively [9]. Drug release profiles for Triogel reached 46% paclitaxel, 46% 17-AAG and 44% rapamycin in 48 h. In contrast, PEG-*b*-PLA micelles carrying paclitaxel, 17-AAG and rapamycin at 6, 6, 3 mg/mL, respectively (Triolimus, micellar solution) released 78% paclitaxel, 91% 17-AAG, and 68% rapamycin in 24 h. Triogel was highly effective in eradicating metastatic peritoneal ovarian tumors in ES-2-luc human ovarian cancer-bearing mice. A single intraperitoneal injection of Triogel carrying paclitaxel, 17-AAG and rapamycin at 60, 60 and 30 mg/kg, respectively, decreased tumor burden from 100% to 6% on the 14th day post treatment. Tumor regression upon a single intraperitoneal injection of Triogel was 70-fold superior than a treatment result of intravenously injected PEG-*b*-PLA micelles carrying paclitaxel, 17-AAG and rapamycin at 60, 60, 30 mg/kg, respectively. This interesting result was presumably due to the different release rates of payloads for hydrogels (slower) and liquid micelles (faster) and duration of drug exposure at the peritoneum of mice (longer residence time for hydrogels than micellar solutions). 

Various research has been done on the use of hydrogels during peritoneal cancer surgery. One area in which hydrogels play a huge role is the possible prevention of post-surgical adhesions from intra-abdominal surgeries. Results through various studies on animals have shown that prevention of post-surgical adhesion is possible with the use of hydrogels. These postsurgical adhesions are not inevitable, still occurring in approximately 67% to 93% of humans who undergo abdominal or pelvic surgery [46,47]. Adhesions commonly occur after gynecological procedures and intraperitoneal surgeries leading to problems such as infertility, intestinal obstruction, chronic abdominal pain, and sometimes requiring re-surgery. During the first three days of post-surgery, abnormal attachments between tissues and the adjacent organs may occur [46,48]. The formation of internal scars that develop from post-surgery, infection, trauma, or radiation has caused complications in many patients. Strategies to prevent the abdominal adhesion formation using thermosensitive polymeric hydrogels have been studied in animals. Polymeric hydrogels, such as PEG-PCL-PEG with its sol-gel-sol property, have been proven to be more biocompatible, biodegradable and nontoxic. Hydrogels provide an effective barrier which will allow the peritoneal tissues and organs to separate long enough during the healing process [49]. The solidity of the gel also contributes to its adhesive characteristics that can be beneficial by adhering to internal organs for the prevention of postsurgical adhesions. Noting that both PEG and PCL are FDA-approved biodegradable and biocompatible materials, PEG-PCL-PEG hydrogels were able to adhere to the affected sites in the peritoneum and were eventually absorbed by the body while the wounds were healing [46,47]. In addition, while evaluating the hydrogel on the injured surfaces that were created on the cecum and abdominal wall of rats, it was found that the hydrogel coat disappeared on the fifth day. However, the residual hydrogel that adhered closely to the damaged surface still appeared on day 7 through day 14 [46,47]. The viscous liquid was completely absorbed by the body by day 14. Thermosensitive hydrogels have also been studied as a novel amphiphilic, multi-drug delivery system for intra-abdominal and peritoneal cancers [9]. Intraperitoneal administration of thermosensitive hydrogels carrying multi-drugs will be useful to prevent the post-surgical adhesion formation and treat residual tumor margins and tissues. 

## 5. Conclusions

In this article, we summarize the types of hydrogels as drug delivery vehicles, various methods to load hydrophobic molecules in hydrogel matrices, and the potential applications of hydrogels in cancer treatment. Hydrogels have shown great potential as a barrier to prevent post-surgical adhesions as well as a support system in the delivery of hydrophilic and hydrophobic drugs to the site of action. Although there have not been many clinical trials over the use of hydrogels for the locoregional treatment of diseases, efforts have been made to tailor hydrogels to impart desirable physicochemical properties, such as biocompatibility and loading capacity for hydrophobic compounds, allowing for optimization of hydrogel-based drug delivery systems.
